# Development of a scale to measure expected concussion reporting behavior

**DOI:** 10.1186/s40621-021-00364-4

**Published:** 2021-12-17

**Authors:** Emily Kroshus, Sarah J. Lowry, Kimberly Garrett, Rachel Hays, Tamerah Hunt, Sara P. D. Chrisman

**Affiliations:** 1 Center for Child Health, Behavior and Development, Seattle Children’s Research Institute, 1920 Terry Ave, Seattle, WA 98101 USA; 2 Department of Health Sciences and Kinesiology, Georgia Southern University, Statesboro, GA P.O. Box 8076, 30460 USA; 3 Department of Pediatrics, University of Washington, 6200 NE 74th St, Ste 110, Seattle, WA 98115 USA

**Keywords:** Concussion, Expected behavior, Measure development

## Abstract

**Background:**

Most concussion education aims to increase athlete self-report of concussive symptoms. Although the population burden of concussion is high, frequency with which this injury occurs on a given sports team in a given season is relatively low. This means that powering concussion education evaluation studies to measure change in post-injury symptom reporting behavior requires what is often a prohibitively large sample size. Thus, evaluation studies are typically powered to measure proximal cognitions. Expected reporting behavior, a cognition that reflects planned *and* reactive decision-making, is a theoretically indicated construct for inclusion in evaluation studies. However, previously no scales were available to measure this construct with demonstrated reliability and validity among youth athletes. The objective of this study was to develop and assess the validity of a brief single-factor scale to measure expected youth athlete concussion reporting behavior (CR-E) in a sample of youth athletes.

**Methods:**

A mixed methods approach was used, including cognitive interviews with youth athletes, and quantitative item reduction and validation. Participants were youth athletes (aged 9–16) from the Seattle metropolitan and rural south-Georgia regions. After refining an initial pool of items using cognitive interviews with a diverse group of youth athletes (*n* = 20), a survey containing these items was administered to youth soccer and football players (*n* = 291). Item reduction statistics and sequential confirmatory factor analyses were used to reduce the initial scale using a randomly selected half of the sample. Then, a final confirmatory factor analysis and validation tests were applied to the other half of the sample of youth athletes. Predictive validation was conducted longitudinally in a separate sample of youth athletes (*n* = 155).

**Results:**

Internal consistency was high (alpha = 0.89), model fit was excellent, validation tests were in the hypothesized directions, and the scale was feasible to use. Using the finalized 4-item scale, we observed that less than one-third of youth soccer and football athletes expect to “always” tell their coach about symptoms of a suspected concussion.

**Conclusions:**

The CR-E measure should be included in future studies evaluating concussion education programming in youth athlete populations.

**Supplementary Information:**

The online version contains supplementary material available at 10.1186/s40621-021-00364-4.

Nearly two million youth athletes in the USA are diagnosed with a concussion each year (Bryan et al. 2016). This number is likely an underestimate of the true prevalence of this injury. Self-report survey data suggest that as many as half of adolescent athletes delay seeking care or never seek care for a suspected concussion (Ferdinand Pennock et al. 2020), which puts them at risk for further injury and prolonged symptoms (Asken et al. 2018). Given that a minority of concussions result in a loss of consciousness and many of the most common concussion symptoms (e.g., headache) are not externally visible, early removal from play for medical evaluation is facilitated by symptom self-report. With a goal of encouraging concussion symptom self-report, most youth sports organizations recommend or require some form of concussion education for athletes (Tomei et al. 2012). Understanding whether these educational initiatives are effective requires measurement tools shown to be valid and reliable in the populations of interest. Ideally, such measures would assess actual concussion reporting behavior. However, behavior is only a relevant outcome if the athlete has been in a situation where concussion reporting was appropriate. In other words, an athlete would need to have sustained an impact to their head or body and experienced symptoms of a possible concussion that were not externally visible (e.g., no loss of consciousness) during the course of the study. Thus, a very large sample size and a longitudinal study design with season-long follow-up would be needed for an evaluation study to be adequately powered using concussion reporting behavior as a primary analytic outcome.

In the short term, and in smaller samples, cognitive precursors to seeking care for a suspected concussion are often used as outcome measures (Kroshus et al. 2015a). Most make the assumption that decisions about concussion reporting are made deliberatively (i.e., employing rational theoretic frameworks such as the Theory of Planned Behavior to guide program evaluations) (Kroshus et al. 2014; Register-Mihalik et al. 2013). Consistent with such frameworks, concussion reporting intentions (a deliberative, planful cognition) are typically employed as a primary outcome measure in evaluation studies (Kroshus et al. 2014; Register-Mihalik et al. 2013, 2017). However, evidence across domains suggests that intentions to perform a given behavior tend to be only moderately correlated with behavior (Webb and Sheeran 2006), an association that has been borne out in extant correlations between concussion reporting intentions and subsequent in-season reporting behavior (Kroshus et al. 2015a).

One explanation for the low correlation between concussion reporting intentions and concussion reporting behavior is that decisions made in sports games or practices are made under conditions of physiologic arousal, fatigue, and normative pressure (Kroshus and Chrisman 2019). In such conditions, risk decisions tend to be emotional or reactive, rather than deliberative and planful (Gerrard et al. 2008). This argues for the use of a dual process approach to understanding concussion reporting behavior, that recognizes the influence of both System I (reactive) and System II (deliberative) thinking (Kroshus and Chrisman 2019; Gerrard et al. 2008). Indeed, Baugh and colleagues found that college football players who scored higher on cognitive reflection (a means of assessing deliberative thinking), were no different from their peers in terms of their concussion reporting (Baugh et al. 2019). Behavioral expectations, or an individual’s self-reported likelihood of engaging in the target behavior in challenging real-world conditions, better capture reactive and emotional decision making as they reflect what an individual thinks is likely to happen, in the “heat of the moment,” rather than what they think would be ideal to have happen (Warshaw and Davis 1985). Consequently, they tend to be more highly predictive of behavior than behavioral intentions (Warshaw and Davis 1985), and may be a more appropriate outcome measure for evaluating concussion education programs.

Problematically, there have as-yet been no reports of rigorous measure development for a standardized scale of concussion reporting expectations for youth athletes. Further, even for extant measures of concussion reporting intention, psychometric properties are weak. Many existing measures of concussion reporting intention contain single items (risking measure instability) or lack contextualization in question framing. A critical challenge with querying youth about their cognitions about concussion reporting is the potential for socially desirable or aspirational responses. Asking athletes to respond with reference to a series of specific naturalistically relevant situations, as is more typical for assessments of behavioral expectations than behavior intentions, is one strategy for mitigating this threat to validity (Urdan and Pajares 2006). In other words, rather than asking them broadly if they intend to or plan to report a suspected concussion, describe a specific, challenging, real-world situation and then ask them what they would be likely to do in that situation.

This study described the development and validation process of an expected concussion reporting behavior scale for pre-high school youth athletes. Most research to-date on concussion reporting behavior and related cognitions has been among college athletes. A key distinction between college athletes and youth athletes is their literacy level; questions that are appropriately worded for a college athlete population may be difficult to interpret or frustrating for a youth athlete. While aiming to develop an appropriate means of assessing concussion reporting in context (i.e., using population-specific scenarios), we also sought to minimize length to allow for feasible use in research conducted in youth sport settings.

## Methods

### Initial item development and refinement

#### Study design and setting

This study was designed to develop and preliminarily validate a brief measure of youth concussion reporting expectations in sport settings. The study involved (1) item and survey development, (2) scale development and preliminary validation using split-half confirmatory factor analysis (CFA) methods in survey data collected at youth soccer and football events in 2018, and (3) subsequent exploratory predictive validation using similar data collected in a separate population the following year.

#### Domain definition

The purpose of the domain is to assess youth athletes’ expected likelihood of reporting a possible concussion in a competitive team sport setting. We operationalized concussion reporting as telling their coach if suspected concussion symptoms were experienced. While there are other adults to whom the athlete could potentially report a concussion, for concussions that occur within the context of games or practices other adults (i.e., parents, medical personnel) are not always present.

#### Item development

Items were generated using a deductive approach which began by reviewing relevant literature and assessing the content and structure of existing scales (Kroshus et al. 2015a, 2014; Register-Mihalik et al. 2013, 2017). Given our goal of developing items regarding specific population-relevant scenarios that would make concussion reporting challenging, we also reviewed literature on determinants of concussion reporting intentions and behavior (Register-Mihalik et al. 2013, 2017; Kroshus et al. 2015b, 2015c; Kerr et al. 2016, 2014; Corman et al. 2019; Cranmer and LaBelle 2018; Wayment and Huffman 2020; Brown et al. 2019). We also drew on items from a prior scenario-based assessment of concussion reporting self-efficacy for young adult athletes (Kroshus et al. 2014). Subsequently, we generated an initial pool of 7 scenario-based items to fit the described domain and population. Reading level was assessed using the Flesch–Kincaid readability test to ensure content was no higher than fifth grade reading level. Four experts in pediatric sports-related concussion reviewed items, affirming domain relevance, representativeness and quality.

#### Cognitive interviews

To assess appropriateness of the initial 7 items to the target domain (content validity) and to examine domain relevance, question clarity, and wording, we conducted cognitive interviews with 20 youth athletes (ages 9–16). Youth were recruited from recreational and competitive soccer and football teams in the greater Seattle, Washington (*n* = 13) and Statesboro, Georgia regions (*n* = 7), using a key-informant led snowball sampling approach. Parent consent and child assent was obtained prior to participation. Trained members of the study team conducted interviews in person. Cognitive interviews used both think aloud and cognitive probing techniques to assess comprehension, retrieval of information, judgment, response formatting and response editing (Collins 2001). The standardized script read: “As you are reading each question, please take a second to (a) check the box next to any items that seem strange, unusual, or difficult to understand and (b) circle specific words or phrases that seem problematic for any reason. Once you’ve finished reading through the survey, I’ll ask you some questions.” The member of the research team administering cognitive interviews used professional judgment, based on prior teaching experience with this age group, to gauge understanding about the task and to answer questions or provide a modified task description as necessary. For each item marked, researchers used probing techniques to assess any problems with questions. To assess comprehension, researchers asked “Can you please tell me the meaning of this item in your own words” and “What about this item is strange, unusual, or difficult to understand?” Research team members were also trained to gauge response latency and asked participants who seemed to struggle on particular questions to probe for comprehension problems and/or identify any response editing (to conform to social desirability). As recommended by DeVellis (DeVellis 2016), cognitive interviews and modifications were iterative. After completing several interviews, we summarized respondent feedback on each item flagged by participants and categorized that feedback based on Knafl’s types of problems in cognitive interviews (limited applicability, unclear reference, unclear perspective, wording or tone) (Knafl et al. 2007). Items that were interpreted correctly by all participants were retained, items flagged by participants as confusing or difficult to understand were modified. No further changes were made to items when respondents did not flag items for discussion and did not seem to the researcher conducting the cognitive interview to struggle with the questions. This modified list of items was brought to the next round of participants. Interviews continued until no further concerns were raised about items. All research procedures were approved by the [redacted for blind review] Institutional Review Boards.

### Survey content and administration

#### Survey content

Questionnaires included the six items developed for the target domain shown in Table 2. Additional items from preexisting validated scales were included for the purposes of convergent validation: a single item assessment of generalized intentions about concussion reporting (Kroshus et al. 2015a; Register-Mihalik et al. 2013), the Weinberger Adjustment Inventory (WAI) Impulse Control subscale (Farrell and Sullivan 2000), and the prosocial subscale of the Pro-social and Anti-social Behavior in Sport (PABBS) measure (Hodge and Lonsdale 2011). We also included questions about prior season concussion reporting behaviors for assessing differentiation by known groups.

### Sample and procedure

Data were collected from two samples: the *main study sample* and the *predictive validation sample*. For the *main study sample*, questionnaires were administered using paper and pencil at soccer and football games or tournaments to a total of 291 youth athletes (ages 9–16) in the greater Seattle, Washington region (*n* = 270) and the Statesboro, Georgia region (*n* = 21) in the fall of 2018. While sample size needs depend in part on the nature of the data and the correlations between items, typical recommendations include 200–300 participants and at least 10 (or even 20) participants per item (DeVellis 2016; Boateng et al. 2018; Morgado et al. 2018; Cabrera-Nguyen 2010). With six initial CR-E items this sample was determined to be a sufficient size for the development of this scale. After development and preliminary validation of the final, reduced scale using split-half CFA methods (described below), we then carried out an exploratory predictive validation in a separate sample (the *predictive validation sample*). A paper-and-pencil questionnaire was completed by soccer and football youth athletes ages 9–14 in the greater Seattle, Washington region at the beginning and end of their Fall 2019 competitive season. The beginning of season questionnaire included the final scale, and the end of season questionnaire asked whether during the season they hit their head and then felt dizzy, had a headache, or felt not quite right (i.e., did they experience a reportable event), and if so, whether they (a) told their coach and (b) stopped playing. Participants were in a separate evaluation of a concussion education intervention. In the present exploratory predictive validation, we included only control condition athletes who had baseline and season-end data (*n* = 118; an additional 37 participants were missing data at one time point); of those, 21 experienced a reportable event (Additional file 1: Table S1).

### Psychometric scale development and validation (Group 1)

#### Item reduction statistics

After questionnaire completion in Fall 2018, we used item reduction statistics to identify potentially problematic items and to inform decisions around item reduction in the next step. For each item we examined floor or ceiling effects (defined as > 70%) and percent missing (Desai et al. 2018). Item-to-total correlations were examined to identify items negatively correlated with the rest of the items, which would indicate that such items were not acting as intended, as the included items were designed to be positively rather than negatively correlated. Item-to-item correlations were used to identify highly correlated pairs of items which could suggest redundancy (> 0.80) (Desai et al. 2018), given desire for a parsimonious scale.

#### Split-half scale development and preliminary validation

We then randomly divided the *main study sample* into equal-sized test and validation samples. We conducted a sequence of confirmatory factor analyses (CFA) on the test sample to identify and omit any poorly performing items in order to develop a more parsimonious scale, carrying out a new CFA with the reduced scale each time an item was removed. We then assessed the goodness of fit of the final model by conducting a CFA on this model in the validation sample. For all CFAs, maximum likelihood estimation was used to obtain parameter estimates along with the Satorra–Bentler standard error estimator which is recommended for smaller samples and non-normal data (Boateng et al. 2018). We elected to use CFA rather than exploratory factor analysis because a single-factor domain was hypothesized a priori (DeVellis 2016; Worthington and Whittaker 2006). Missingness was handled throughout by omitting those observations missing responses to some or all CR-E questions; this approach was used due to low degree of missingness (3.8–5.5%). An initial model was fit using all 6 CR-E items, and then model fit was assessed using Satorra–Bentler-adjusted Chi-squared tests, root-mean-square error of approximation (RMSEA), the comparative fit index (CFI), and the Tucker–Lewis Index (TLI) (Boateng et al. 2018; Cabrera-Nguyen 2010). Items were removed one at a time if they were deemed to be problematic based on item reduction statistics, factor loadings, conceptual and/or construct appropriateness, and the model was re-fit and the new fit statistics were then examined. This process continued until the reduced scale demonstrated good model fit. The goodness of fit of this final reduced scale was then re-assessed independently by fitting the final model using the separate validation half of the *main study sample* (DeVellis 2016).

#### Scoring the scale

Scale score was calculated as the average of the 4 retained items (possible range: 0–4) and was set to missing for participants without responses to more than 2 of the 4 items.

#### Reliability tests

To assess the internal consistency of the scale, we calculated Cronbach’s alpha of the final scale in the validation sample, defining an optimal value as 0.8–0.9 (Morgado et al. 2018) or 0.8–0.95 (Boateng et al. 2018).

#### Validation tests

We tested a priori hypotheses about the direction and strength of correlation between the final CR-E score and specified scales and reported behaviors in the validation half of the *main study sample*. Strength of correlation was assessed via Pearson correlations except in the case of binary variables, for which point-biserial correlations were calculated. ***Concurrent validation,*** or whether scale scores are associated with legacy measures assessed at the same time (Boateng et al. 2018), was gauged by assessing correlation between CR-E scale score and self-reported behaviors among the subset of youth with a recent possible concussion or head injury, i.e., those who reported actually having hit their head in the past 3 months and then feeling dizzy, having a headache, or feeling not quite right. We expected moderate positive correlations between the new instrument and recent behavior of reporting a head injury to someone, (and moderately strong inverse correlation with duration of time they continued playing after the injury)**. *Convergent validation*** was assessed as the correlation with a single item assessment of generalized intentions about concussion reporting, as used in extant literature (Kroshus et al. 2015a; Register-Mihalik et al. 2013). While behavioral expectations have some similar cognitive antecedents but differ in other key ways, we expected a low to moderate correlation positive with the new scale. We also expected the new scale to be weakly to moderately correlated with two other constructs theoretically related to concussion reporting: impulse control (i.e., self-regulation to facilitate volitional behavior), and pro-social behavior (i.e., rule following). We anticipated higher CR-E scores to be moderately correlated with higher impulse control, as measured by the Weinberger Adjustment Inventory (WAI) Impulse Control subscale (Farrell and Sullivan 2000), as well as with higher prosocial behavior, as measured by the prosocial subscale of the Pro-social and Anti-social Behavior in Sport (PABBS) measure (Hodge and Lonsdale 2011). ***Differentiation by “known groups”*** was assessed as the correlation between the new measure and history of previously diagnosed concussion. Based on prior literature (Register-Mihalik et al. 2017; Kroshus et al. 2020; O’Connor et al. 2020 Apr 2), we expected that those who had previously had a concussion would be less likely to expect to report a future concussion.

#### Predictive criterion validation

In the *exploratory predictive validation sample* of soccer and football athletes ages 9–14 described above (*n* = 118), we examined the association between CR-E score at the start of the season, and behaviors reported at season end, among players who had sustained a blow to the head during the season (*n* = 21). We hypothesized that those reporting safer behaviors at the end of the season would also have a higher baseline mean CR-E score compared to those reporting less safe behaviors. The behaviors of interest included whether they kept playing, and whether they told someone (or reported that someone already knew) that they had hit their head.

## Results

### Cognitive interviews

Participant feedback from cognitive interviews is summarized in Table 1. Overall, participants had little feedback about the wording of the questions; the main problem was with the question prompt. Originally it said “If I think I had a concussion, I would tell my coach…” Participants expressed uncertainty about the definition of a concussion, meaning that their responses were contingent on how they defined a concussion (e.g., Does it require a loss of consciousness, or are relatively less acute symptoms also indicative of a potential concussion?). Addressing this problem, we changed the prompt to “If I felt dizzy after a bump or hit to the head, I would tell my coach…” Subsequent feedback from participants about the uncertain temporality of telling their coach led to us changing the wording to “if I felt dizzy after a bump or hit to the head, I would tell my coach right away…”. We made one change to the question “…even if my team is losing” to soften the tone. Athletes who played for less competitive teams remarked that the wording was “mean” and did not reflect their team culture. Based on this feedback, researchers changed this question to “even if it was a close game.” We similarly dropped one other item “... even though I am a top player” to allow for personal resonance across a range of competitive levels and individual ability levels. Participants considered the revised question stem to be clear and appropriate. While we explored the possibility of providing a more comprehensive list of concussion symptoms to participants, we opted to include a single representative symptom (dizziness). Ultimately, the longer list of symptoms was viewed as too burdensome and not something that would increase the validity of the question.

**Table 1:** Summary of feedback from cognitive interviews

Original item	Feedback theme (n)	Example(s)	Modification	Final version
(stem) If I think I had a concussion I would tell my coach… (v1)	Unclear reference (3)	“…my answers depend on the how sure you are and the severity of the concussion. If it wasn't very severe and you weren't sure, you probably wouldn't say something, especially if it's the championship game and the team is counting on me to play.” “If I knew I'd say strongly agree across the board. I based my answers off of that I'm pretty sure I have a concussion. For me it has to do with severity.”	“If I felt dizzy after a bump or hit to the head, I would tell my coach…” (v2)	“If I felt dizzy after a bump or hit to the head, I would tell my coach right away…”
	Unclear reference (1)	“I would play but if I didn't feel worse, I may not tell coach right away.”	“If I felt dizzy after a bump or hit to the head, I would tell my coach right away…” (v4)	
…even if the team was counting on me to play	No feedback	–	Unmodified	–
…even during a championship game	No feedback	–	Unmodified	–
…even if I really wanted to keep playing	No feedback	–	Unmodified	–
…even if my team is losing	Wording/tone	Respondents who played for less competitive teams flagged questions like this in other scales and remarked that the negative wording was “mean.”	“…even if it is a close game”	“…even if it is a close game”
…even if my team will be down a player	No feedback	–	Unmodified	–
…even though I am a top player	Other	Dropped to address concerns about competitive level	Dropped	–
“…even if my parent/guardian said I’m fine to play”	No feedback	–	Unmodified	–

### Demographic characteristics

Of the 291 participants in the *main study sample*, 17% played football and 83% played soccer; 50% were male; and participant age ranged from 9 to 16 years (Table 2). Approximately 62% identified as White, and 10–12% each identified as Asian, other race, or reported more than one race; fewer than 5% each identified as Black or Native Hawaiian/Pacific Islander. Approximately 17% identified as Hispanic, with an additional 15% unsure of their ethnicity. One in five (21%) reported ever having been diagnosed with 1 or more concussions. In the *exploratory predictive validation sample,* of the 21 participants who during the season hit their head and then felt dizzy, had a headache, or felt not quite right (i.e., participants who experienced a reportable event), 71% played football and 29% played soccer; 85% were male; and participant ages ranged from 10 to 14 years. Race and ethnicity data were missing for approximately a third of the sample but approximately 20% of those with known ethnicity were Hispanic. Approximately 40% of those with known race were White, 29% Black, 20% reported multiple races and 7% each reported Asian and Native Hawaiian/Pacific Islander.

**Table 2:** Demographic characteristics of participants in survey validation (Group 1)

	*N*	%
**Location**		
Georgia	21	7.2
Seattle	270	92.8
**Sport**		
Soccer	242	83.2
Football	49	16.8
**Gender**		
Male	139	50.4
Female	130	47.1
Prefer not to answer	7	2.5
**Age (years)**		
9	18	6.5
10	36	13
11	50	18.1
12	30	10.8
13	93	33.6
14	40	14.4
15	6	2.2
16	4	1.4
**Race**		
Caucasian or White	161	62.2
African American or Black	11	4.2
Asian-American	32	12.4
Native Hawaiian/Pacific Islander	3	1.2
Other	25	9.7
More than one race reported	27	10.4
**Mexican, Hispanic, or Latin American descent**		
No	187	67.8
Yes	47	17.0
I don't know	42	15.2
**Number of times diagnosed with a concussion**		
0	159	79.1
1	24	11.9
2	12	6.0
3	2	1.0
4	4	2.0
5 +	0	0

Numbers may not sum to total (291) due to missing values

### Item reduction statistics

Variability was good with responses distributed across all 5 categories for all 6 items, increasing towards the higher response values for all items; no more than 13% or 32% of respondents answering the lowest or highest response categories respectively (Table 3). Data missingness was low, ranging from 3.4 to 5.5% missing across the CR-E items (Table 3). Of the 291 participants, 269 (92%) provided answers to all 6 CR-E questions. Ten (3%) were missing 5 of the 6 questions, 2 were missing 4, 1 was missing 2, 1 was missing 1 (each < 1%), 9 (3%) were missing all 6, all of which were from the same survey round. Item-to-item correlations ranged from 0.60–0.85; item-to-total correlations ranged from 0.83–0.92 (Table 4).

**Table 3:** Survey item, number of respondents, distribution of responses, reasons for exclusion (if excluded from final measure)

ICR Survey Item	Number of respondents	Distribution of responses (%)						Mean (SD)	Reasons for Exclusion
Question stem: If I felt dizzy after a bump or hit to the head, I would tell my coach right away…		Never (%)	Rarely (%)	Sometimes (%)	Often (%)	Always (%)	Missing (%)		
1. …even if the team was counting on me to play	282	7	9	23	25	32	*3*	2.68 (1.24)	(Retained)
2. …even during a championship game	281	8	16	24	22	27	*4*	2.44 (1.28)	Conceptual overlap with #1; *r* = 0.81
3. …even if I really wanted to keep playing	276	6	13	25	24	27	*6*	2.54 (1.22)	(Retained)
4. …even if it was a close game	280	9	14	21	25	28	*4*	2.52 (1.29)	(Retained)
5. …even if my parent/guardian said I'm fine to play	277	8	16	20	21	31	*5*	2.52 (1.33)	Not central to the construct (more normative)
6. …even if my team will be down a player	279	13	19	22	16	26	*5*	2.26 (1.39)	(Retained)

**Table 4:** Item-to-Item and Item-to-Total Correlations (test sample)

Item	1	2	3	4	5	6	Total
1	1.00						0.87
2	0.81	1.00					0.91
3	0.79	0.79	1.00				0.92
4	0.76	0.79	0.85	1.00			0.92
5	0.60	0.68	0.71	0.71	1.00		0.83
6	0.68	0.76	0.76	0.79	0.72	1.00	0.88

### Split-half scale development CFAs

Sequential CFAs were conducted on the test sample, and model fit statistics assessed at each round (Table 5). Fit statistics for the full set of 6 items were relatively good with the exception of RMSEA (Table 5). Item reduction statistics did not reveal poorly performing items, but item 5 was removed as it was deemed less central to the construct. This resulted in borderline Satorra–Bentler-adjusted Chi-squared p-value and RMSEA (Table 5), both of which were resolved after the additional removal of item 2 due to conceptual overlap with item 1(*r* = 0.81). Goodness of fit of the final model, which included items 1, 3, 4, and 6, was then assessed by conducting a final CFA on the validation half of the *main study sample*, and satisfactory model fit statistics were observed (Table 5).

**Table 5:** Model fit statistics for CFA at each step of scale development

Round	Model	Chi-squared p-value	Degrees of freedom	RMSEA	CFI	TLI
1	All 6 original CR-E items	0.070	9	0.075	0.987	0.978
2	Remove item 5	0.047	5	0.094	0.986	0.973
3	Remove item 2	0.255	2	0.051	0.998	0.994
4	Validation Sample	0.660	2	< 0.001	1.000	1.000

RMSEA: root-mean-square error of approximation; CFI: comparative fit index, TLI: Tucker–Lewis Index. Satorra–Bentler scaled model fit indices were used, as is recommended for models with non-normal variables and smaller sample sizes. (Boateng et al. 2018)

Thresholds used for good fit: Chi^2^ *p* > 0.05; (Cabrera-Nguyen 2010) RMSEA ≤ 0.06, (Hu and Bentler 1999) CFI > 0.95, TLI > 0.95 (Schreiber et al. 2006)

### Assessment of reliability and validity

#### Internal consistency reliability

Cronbach’s alpha of the scale using the validation sample was 0.89.

#### Concurrent validation

Thirty participants in the validation half of the *main study sample* indicated that during the previous 3 months they hit their head and then felt dizzy, had a headache, or felt not quite right. When they were asked about how long they continued playing, 3 reported they stopped right away, 9 within a few minutes, 6 in the same half, and 11 played most or the rest of the game. There was a weak inverse association between how long they continued playing and CR-E score (Pearson’s *r* = − 0.275). Most (*n* = 20) reported that they did tell someone about how they were feeling, with 7 reporting that they did not, and 3 missing a response; telling someone about how they were feeling correlated moderately with CR-E score (point-biserial correlation *r* = 0.451).

#### Convergent validation

We observed a moderate correlation with the single item concussion reporting intention measure (Pearson correlation *r* = 0.45, *n* = 134). We observed a small, positive correlation between CR-E score and WAI Impulse Control subscale score (Pearson correlation *r* = 0.26, *n* = 105) and between CR-E score and PABBS Pro-social subscale score (Pearson correlation *r* = 0.10, *n* = 109). As hypothesized, individuals with greater impulse control and greater pro-social orientation were more likely to expect to report a future concussion.

#### Differentiation by known groups

There was a small, negative correlation between CR-E score and history of having one or more previously diagnosed concussion (point-biserial *r* = 0.14) and the number of concussions previously diagnosed (Pearson *r* = − 0.11) (*n* = 131); as hypothesized, individuals with previously diagnosed concussions were less likely to expect to report future concussions.

#### Predictive criterion validation (exploratory)

In the *exploratory predictive validation sample* of soccer and football athletes who had hit their head during the season and then felt dizzy, had a headache, or felt not right, those who reported that they did NOT keep playing after the injury (safe behavior) had a slightly higher baseline CR-E score compared to those who did keep playing, but it was not statistically significant: 2.8 (95% CI 1.5–4.0) vs 2.6 (95% CI 1.8–3.3). Only 21 athletes were included in this analysis due to the rare outcome. Similarly, those who reported either telling someone, or that someone already knew, had a higher baseline CR-E score as well 2.9 (95% CI 2.1–3.7) vs 2.1 (95% CI 1.0–3.3).

### Description of expected concussion reporting behavior

In the validation sample, for each of the final scale item between one-quarter and one-third of respondents indicated they would “always” tell their coach right: “even if the team was counting on me to play” (32%), “even if I really wanted to keep playing” (27%), “even if it was a close game” (28%), and “even if my team would be down a player” (26%). Item and full-scale scores were normally distributed, indicating a modal behavior or “sometimes” or “often” engaging in the desired safe behavior.

## Discussion

Addressing the current lack of an age-appropriate measure of expected concussion reporting behavior, the present study reports the development of a parsimonious 4-item single-factor scale. We followed a standard stepwise approach to development and validation, producing a final scale with excellent model fit statistics that were reproduced in the separate validation sample, and that demonstrated high internal consistency reliability. Convergent, concurrent, and discriminant validation results were in the anticipated directions. Importantly, youth athletes were engaged early in the process of developing the scale, providing feedback on item clarity and relevance. Based on their input we made critical adjustments including reducing the reading level, adjusting language to soften the tone and making items more universally appropriate across youth sports.

The CR-E measure can contribute to improved quality of concussion education program evaluations. Broadly, we note that for such evaluations to be useful, they should assess reporting behavior across a sports season or other unit of time during which behavior change could reasonably be expected to occur. If this is not feasible due to sample size limitations, constructs most closely associated with reporting behaviors should be assessed. This means concussion knowledge is not a sufficient outcome as it is at best weakly correlated with reporting behavior (Kroshus et al. 2015a; Register-Mihalik et al. 2017; Rawlins et al. 2020)). Some concussion education program evaluations assess reporting intentions as a proxy for reporting behavior (Schmidt et al. 2020; Kneavel et al. 2020), however we note that intentions are a planful and deliberative construct and tend to be only moderately correlated with behavior (Kroshus et al. 2015a). Reporting expectations are more theoretically consistent with the rational *and* reactive/emotional ways in which concussion reporting decisions are likely made (Kroshus and Chrisman 2019). Thus, CR-E is a theoretically indicated approach to evaluating concussion education programming for youth athletes when it is not feasible to assess behavioral outcomes.

Responses to items retained in the final 4-item scale indicate a need to improve expected reporting behavior. Across all items, fewer than half of participants indicated that would “always” tell a coach about possible symptoms. Self-report of suspected concussion symptoms is particularly important at the youth level as youth sports are unlikely to have medical staff on the sideline (as compared to collegiate or professional sport), and thus have less external oversight as to whether an injury that requires medical evaluation. Structural changes to youth sport may be the most effective means to facilitate concussion identification and removal from play: prior research finds that more concussions are identified on high school teams that have an athletic trainer present at games (Kroshus et al. 2017). However, such initiatives are often cost prohibitive, limiting their likelihood of being enacted as a mandate at a policy level or equitably and consistently implemented volitionally by youth sport organizations. Thus, most states and sports organizations focus on concussion education as a feasible and low cost approach to improving concussion reporting. Critically, having appropriate means of evaluating existing and novel approaches to concussion education will facilitate the process of identifying interventions that positively impact youth concussion reporting behavior (Kroshus et al. 2020). The CR-E measure can also be used to evaluate the impact on expected athlete behavior of other education initiatives oriented at creating sports organization, team, and family cultures that are supportive of concussion reporting (i.e., coach education, parent education).

### Limitations

Participants in the development and validation process were youth football and soccer athletes in two regions of the country. However, we note that this sample was unbalanced by region. We caution that the measure may not have similar psychometric properties in other sport settings or age groups and recommend further testing of this instrument in other populations of youth athletes. We note that the present study did not assess test–retest reliability given anticipated change across the competitive season. We also only conducted confirmatory, rather than exploratory, factor analyses.

## Conclusions

The measure developed in the present study is brief and feasibly included in survey-based assessments of the effectiveness of concussion education programming. We encourage sport governing bodies, high school athletic associations, and other organizations involved in mandating or adopting concussion education to search to see if the program they are employing has demonstrated effectiveness in improving concussion reporting behavior or expected behavior. In the absence of this information, we encourage youth sports organizations to partner with trained evaluators to administer brief surveys before and after concussion education is delivered, including the present scale.

## Supplementary Information

**Additional file 1:** Demographic characteristics of predictive validity sample.

## Data Availability

The datasets used during the current study are available from the corresponding author on reasonable request.
