# Supplementary material for: Development of a scale to measure expected concussion reporting behavior
Source: Inj Epidemiol. 2021 Dec 17;8:70. doi: 10.1186/s40621-021-00364-4 (PMC8684105; doi:10.1186/s40621-021-00364-4)
Supplement: Supplementary file 1 — Additional file 1: Demographic characteristics of predictive validity sample. [file 40621_2021_364_MOESM1_ESM.docx]

**Supplementary table. Demographic characteristics of predictive validity sample**

|  | **Participants who had baseline and end-of-season data** | **Of those, participants who experienced a blow to the head during the season** |
| --- | --- | --- |
|  | **n=118** | **n=21** |
| Soccer (vs Football) |  |  |
| Football | 69 (58.5%) | 15 (71.4%) |
| Soccer | 49 (41.5%) | 6 (28.6%) |
| Male gender |  |  |
| Female | 28 (23.7%) | 3 (14.3%) |
| Male | 90 (76.3%) | 18 (85.7%) |
| Child Age (years) |  |  |
| 9 | 4 (3.4%) | 0 (0%) |
| 10 | 19 (16.1%) | 2 (9.5%) |
| 11 | 27 (22.9%) | 5 (23.8%) |
| 12 | 32 (27.1%) | 6 (28.6%) |
| 13 | 27 (22.9%) | 6 (28.6%) |
| 14 | 3 (2.5%) | 1 (4.8%) |
| (missing) | 6 (5.1%) | 1 (4.8%) |
| Child Race |  |  |
| White | 62 (52.5%) | 6 (28.6%) |
| Black | 5 (4.2%) | 4 (19.0%) |
| Asian | 11 (9.3%) | 1 (4.8%) |
| Native Hawaiian/Pacific Islander | 1 (0.8%) | 0 (0%) |
| Other | 8 (6.8%) | 1 (4.8%) |
| Multiple races specified | 13 (11.0%) | 3 (14.3%) |
| (missing) | 18 (15.3%) | 6 (28.6%) |
| Hispanic ethnicity |  |  |
| No | 70 (59.3%) | 11 (52.4%) |
| Yes | 13 (11.0%) | 3 (14.3%) |
| (missing) | 35 (29.7%) | 7 (33.3%) |
